# Physiological Functions of the Cello-Oligosaccharides Binding CebE in the Pathogenic *Streptomyces* sp. AMCC400023

**DOI:** 10.3390/microorganisms12030499

**Published:** 2024-02-29

**Authors:** Qiuyue Li, Jiawen Chang, Peiwen Lv, Junxia Li, Yuxia Duan, Dandan Tian, Fei Ge, Gaoya Su, Fengjie Nie, Zheng Gao, Chunyu Yang, Bo Zhou

**Affiliations:** 1 College of Life Sciences, Shandong Agricultural University, Tai’an 271018, China; tangli83_1998@163.com (Q.L.); a270092516@126.com (J.C.); 17816399978@163.com (J.L.); d13589684295@163.com (Y.D.); 15666767992@163.com (D.T.); 19819677027@163.com (F.G.); urnotsgy@sina.com (G.S.); gaozheng@sdau.edu.cn (Z.G.); 2 State Key Laboratory of Microbial Technology, Institute of Microbial Technology, Shandong University, Qingdao 266237, China; 201812506@mail.sdu.edu.cn; 3 Research Center of Agricultural Biotechnology, Ningxia Academy of Agricultural and Forestry Sciences, Yinchuan 750002, China; niefengjie@163.com

**Keywords:** pathogenic *Streptomyces*, cello-oligosaccharides binding protein, binding affinity, sporulation

## Abstract

Potato common scab, an economically important disease worldwide, is caused by pathogenic *Streptomyces* strains mainly through the effects of thaxtomin. The cello-oligosaccharides binding protein CebE is proposed as a gateway to the pathogenic development of *Streptomyces scabiei*. In this study, two functional CebE encoding genes, *GEO5601* and *GEO7671*, were identified in pathogenic *Streptomyces* sp. AMCC400023. With a higher binding affinity towards signal molecules, the deletion of *GEO5601* severely impaired thaxtomin-producing capacity and reduced the strain’s pathogenicity. Transcriptional analysis confirmed that CebE^5601^ is also responsible for the import and provision of carbon sources for cell growth. With lower binding affinity, the pathogenicity island (PAI)-localized CebE^7671^ may assume a new function of mediating the biological process of sporulation, given the significantly impaired formation of Δ*GEO7671* spores. The mechanisms of action of CebE proteins unraveled in *Streptomyces* sp. AMCC400023 will help pave the way for more effective prevention of the potato common scab disease.

## 1. Introduction

*Streptomyces* are well-known soil saprophytes that actively participate in the recycling of nutrients in the environment, produce a great variety of secondary metabolites, and induce beneficial activities in plants, such as growth promotion and disease resistance [1]. However, in the last decades, a particular group of *Streptomyces* species was found to harbor the ability to infect important commercial root and tuber crops, including potatoes, beets, radishes, sweet potatoes, and peanuts [2]. It has been confirmed that pathogenic *Streptomyces*, including *Streptomyces scabiei*, *S. acidiscabies*, and *S. turgidiscabies,* can cause raised, pitted, or superficial scab lesions on the potato surface [3,4]. Such potato common scab significantly decreases crop quality and leads to severe economic losses [5].

Thaxtomin is a family of phytotoxins produced by pathogenic *Streptomyces* and is known as the major causative agent of potato common scab [6,7]. These compounds can induce plant cell hypertrophy, root growth retardation, and tissue necrosis at a nanomolar concentration by inhibiting the cellulose synthase complex [8,9,10]. Notably, almost all pathogenic *Streptomyces* have a pathogenicity island (PAI) composed of two regions. The toxin region (TR) harbors the thaxtomin biosynthetic gene cluster, and the colonization region (CR) contains two genes of *nec1* and *tomA*, which encode secreted proteins known or suspected to act as virulence factors [11,12,13]. A previous study has experimentally confirmed the transferability of the PAI among *Streptomyces* species, which might account for the emergence of new pathogenic *Streptomyces* and the spread of potato common scab [14].

Cello-oligosaccharide is the main product of cellulose degradation and is abundant in nature. Some saprophytic *Streptomyces* species specifically utilize cellulose as their carbon source, and employ extracellular cellulolytic enzymes and an inducible ATP-dependent uptake system specific to cello-oligosaccharide [15]. Interestingly, cellobiose and cellotriose were found to possess a function other than providing energy in *S. scabiei* strains. As a major molecule released in the expansion period of plant tissues, cellotriose is proposed as the best inducer and trigger of the synthesis of virulome, including thaxtomin [16,17]. It has been authenticated that the cello-oligosaccharide transport system CebEFG-MsiK is essential for the induction of thaxtomin in pathogenic *Streptomyces* strains [4,18,19]. As the core component of this transporter system, the solute-binding protein CebE is responsible for recognizing and binding the cello-oligosaccharide substrates [20,21]. In contrast to the non-pathogenic *Streptomyces* strains, the transcriptional repressor CebR not only regulates cello-oligosaccharides uptake by binding with cellobiose or cellotriose, but is also involved in unlocking thaxtomin synthesis when binding with the upstream sites within the thaxtomin biosynthetic cluster. CebR has two binding sites (*cbs*) within the thaxtomin gene cluster; one located upstream of *txtR*, the cluster-situated regulator, and another within *txtB*, the peptide synthetase gene (thaxtomin synthetase). As the ligands of CebR, the binding of cellobiose and cellotriose can inhibit its DNA-binding activity, thereby allowing the transcription of *txtR* and inducing thaxtomin biosynthesis [19,22,23,24].

The special regulation mode of thaxtomin biosynthesis confirms the significant contributions of cello-oligosaccharides in *Streptomyces* pathogenicity; CebE was named the “doorway” in pathogenic *Streptomyces* due to its extracellular binding of cello-oligosaccharides and transmission of the signal to the cells, and consequently triggering the synthesis of arsenal thaxtomins [25]. The investigation of CebE can enhance our understanding of the pathogenic mechanism and elucidate how pathogenic *Streptomyces* establishes a connection between sensing extracellular plant material and initiating its virulent behavior [25], thereby establishing a solid foundation for future biological control. In accordance with some pathogenic *Streptomyces*, the common scab representative species *S. scabiei* 87–22 possesses three homologs of CebE, among which *SCAB57751* was initially identified as the major contributor to potato scab, exhibiting a nanomolar range *K*_D_ value towards cellotriose and cellobiose [25]. Recently, the physiological functions of the other two CebE proteins, *SCAB2421* and *SCAB77271*, were also investigated. The deletion of *SCAB2421* caused a moderately attenuated virulence phenotype, while the loss of the PAI-located CebE (*SCAB77271*) had no significant influence on strain growth or virulence capacity [26].

To date, many pathogenic *Streptomyces* species have been isolated, and diverse sets of CebEFG-Msik ABC transporters have been annotated in different pathogenic *Streptomyces* strains. Whether these CebEFG-MsiK ABC transporters play a role in virulence is of great interest and remains to be elucidated. However, the primary research focus on CebE primarily lies within strain *S. scabiei* 87–22, with a scarcity of related studies concerning other strains. Therefore, it is interesting to explore whether other mechanisms exist in other pathogenic *Streptomyces* strains. The pathogenic *Streptomyces* sp. AMCC400023 was previously isolated from potato common scab in Hebei province, China [27]. By possessing the well-known PAI component in its genome, all evidence from radish seedling assay, potted back experiment, and thaxtomin production confirmed *Streptomyces* sp. AMCC400023 is a virulent pathogen causing potato scab. As revealed by genome-based phylogenetic analysis, average nucleotide identity (ANI value is 88%), and in silico DNA-DNA hybridization (isDDH), strain AMCC400023 has a relatively close relationship with *S. scabiei* at the species level. However, comparative genomic analysis in the Virulence Factors of Pathogenic Bacteria Database identified 60 unique virulence-associated genes in the genome of *Streptomyces* sp. AMCC400023 when compared to *S. scabiei* 87–22. Also, unlike the three functional clusters for cello-oligosaccharides uptake in *S. scabiei* 87–22, two CebEFG-MsiK clusters were identified in the genome of AMCC400023. In the present study, we found that these two CebEs perform diverse physiological roles in strain growth and pathogenesis by comparing their binding affinities with cello-oligosaccharide substrates and pathogenesis of CebE-deficient strains, as well as transcriptomic responses in the presence of cellobiose.

## 2. Materials and Methods

### 2.1. Bacterial Strains, Plasmids, and Growth Conditions

Bacterial strains and plasmids used in this study are described in Table 1. *Streptomyces* strains were grown in Gause’s No. 1 medium [28], oat bran broth (OBB) medium [29], thaxtomin-defined medium (TDM) that was supplemented with 7 g L^−1^ cellobiose (TDMc) medium [16], or mannitol soya flour (MS) medium [30] at 28 °C. *E. coli* strains were grown in Luria-Bertani (LB) medium at 37 °C. When required, the medium was supplemented with ampicillin (100 μg mL^−1^), kanamycin (50 μg mL^−1^), chloramphenicol (30 μg mL^−1^), nalidixic acid (25 μg mL^−1^), or thiostrepton (20 μg mL^−1^). Cellobiose and cellotriose were biotech grade and purchased from MACKLIN.

### 2.2. Bioinformatic Analysis

MAFFT was used for multiple sequence alignment and conserved sites were selected to construct the NJ phylogenetic tree using the JTT substitution model, with a bootstrap value of 1000. The CebR binding sites were predicted using the PRODORIC tool.

### 2.3. Protein Expression and Purification

Genomic DNA was isolated by the Tiangen DNA extraction kit (Tiangen Biotech Co., Ltd., Beijing, China). The hydrophobic region of the sequence was analyzed using SignalIP 6.0 and DeepTMHMM v0.0.10. The open reading frame encoding GEO1108/GEO5601/GEO7671 without the hydrophobic region was amplified by PCR using the genomic DNA of *Streptomyces* sp. AMCC400023 as the template and primers listed in Table S3. The amplified fragment was ligated with pEASY-Blunt and transformed into *E. coli* DH5α for DNA sequencing. Then, the gene fragment was ligated with pET28a and transformed into *E. coli* BL21(C43) competent cells. For protein expression, the *E. coli* transformants were cultured in the LB medium at 37 °C containing 50 μg mL^−1^ of kanamycin until the growth reached an absorbance at 600 nm (OD_600nm_) of 0.6. Then, the His-tagged CebE was induced overnight (12 h) at 16 °C by a final concentration of 0.5 mM isopropyl-β-D-thiogalactopyranoside (IPTG). Cells were collected by centrifugation, resuspended in the lysis buffer (25 mM Tris-HCl, 150 mM NaCl, pH 8.0), and lysed using a high-pressure cell crusher. After centrifugation, the soluble proteins were loaded onto the pre-equilibrated Ni-NTA column and eluted with 250 mM imidazole. The cell lysis and purified proteins were verified by SDS-PAGE.

### 2.4. MST Assays

Microscale thermophoresis (MST) assays measurements were performed in triplicate at 25 °C, with 90% excitation power and medium MST power, on the Monolith NT.115 system. The protein was labeled with a red fluorescent dye and applied at a final concentration of 800 nM. The cellobiose and cellotriose solutions were two-fold diluted 16 times, with an initial concentration of 100 μΜ for CebE^5601^ and 270 mM for GebE^7671^. The labeled protein was then added to a dilution solution of the cello-oligosaccharides. Samples were filled into standard-treated capillaries for measurement. All the data were analyzed with MO.Affinity Analysis v2.3 software provided by the manufacturer.

### 2.5. Construction of CebE-Deficient Mutants

The in-frame deletion *Streptomyces* mutants were constructed by homologous recombination technology. DNA fragments about 1 kb upstream and downstream of *GEO5601* or *GEO7671* were amplified, using primers listed in Table S3. The PCR products were purified and ligated into the shuttle plasmid pJTU1278 to generate the pJTU1278-5601 and pJTU1278-7671 plasmids. Then, the constructed plasmids were introduced into *E. coli* ET12567 by transformation. After sequence verification, *E. coli* ET12567 strains carrying pJTU1278 plasmids (*E. coli* ET12567/pJTU1278-5601 or *E. coli* ET12567/pJTU1278-7671) were cultured in LB medium containing antibiotics (100 μg mL^−1^ Ampicillin, 50 μg mL^−1^ Kanamycin, 30 μg mL^−1^ Chloramphenicol) until OD_600nm_ reached 0.4–0.6. The cells were collected by centrifuging at 8000× *g* and washed with fresh LB medium, then collected and suspended in 500 μL of sterile water. For the preparation of *Streptomyces* competent cells, the spores of *Streptomyces* sp. AMCC400023 were collected from the OBB plates and placed in 5 mL TES buffer (10 mM Tris HCl, 1 mM EDTA, 0.1 mM SDS). After heat shock and ice bath, 5 mL YT medium was added and cultured at 37 °C for 2.5 h. Then, the spores were collected by centrifugation and resuspended with sterile water, mixed with the cultures of *E. coli* ET12567/pJTU1278-5601 or *E. coli* ET12567/pJTU1278-7671, and spread on the MS plates. After culturing at 30 °C for 2–3 d, 1 mL of antibiotic solution (0.25 mg mL^−1^ thiostrepton and 0.5 mg mL^−1^ nalidixic acid) was added and then incubated for 16 h. The colonies were transferred to MS medium supplemented with 0.25 mg mL^−1^ tryptophan and 0.5 mg mL^−1^ nalidixic acid, then cultured at 30 °C until confirmed by PCR amplification and sequencing, using primers listed in Table S3.

### 2.6. Plant Virulence Assays

To assess the virulence phenotype of *Streptomyces* sp. AMCC400023 and mutants, an in vitro radish seedling assay was performed. Radish seeds were surface sterilized with 75% ethanol for 8–10 min and rinsed 3–4 times with sterile distilled water. The seeds were allowed to germinate for about 24–48 h in the dark, in a petri dish containing a moistened filter paper. Homogenously germinated seeds with a similar shoot length were placed into a glass tube of 1% agar water, with 3 seeds per tube. An appropriate amount of spore suspension with an OD value of 1.0 was inoculated in 100 mL of OBB for 5 d, then 200 µL of the culture was inoculated with newly sprouted radish seedlings, and the same amount of OBB medium was used as blank control. The tubes were incubated at 24 °C for 6 d, with intermittent light (16 h light and 8 h dark). Each experiment was performed in triplicate. To perform statistical analysis, we employed the Kruskal-Wallis test and Mann-Whitney test. Additionally, we applied the Benjamini-Hochberg procedure to correct the false discovery rate (FDR).

The potato pot trial was conducted from August to December 2020 at Shandong Agricultural University. Fifteen healthy potato tubers of variety Favorita were selected, surface sterilized with 1% NaClO for 5 min, and rinsed 3 times with sterile distilled water. After germination, potato seedlings with a similar shoot length were planted in a 35 × 30 (cm) plastic pot with 10 kg of soil. *Streptomyces* sp. AMCC400023 and the mutant derivatives were cultured on Gause’s No. 1 medium at 28 °C for 10 d. Spores were collected with sterile distilled water and inoculated in OBB media at 28 °C for 7 d. The cultures were adjusted to the same OD value by fresh OBB, then inoculated during the potato expansion period and the final concentration of the *Streptomyces* strains was 10^6^ CFU/cm^3^. Each treatment was repeated in fifteen pots. Samples treated with OBB medium and sterile distilled water worked as a blank control. After 90 d of cultivation, the disease index of potatoes was calculated.

Disease index (percentage) = Σ(incidence level × number of corresponding grades)/(the highest incidence level × total number in this survey) × 100.

### 2.7. Thaxtomin Quantification

With similar spore inoculation, *Streptomyces* sp. AMCC400023 and its mutants were inoculated into OBB medium that was prepared in the same batch, and cultured at 28 °C and 180 rpm for 6–7 d. The cultures were filtered, the supernatant was extracted 3 times with an equal volume of ethyl acetate for 12 h, and then an appropriate amount of anhydrous sodium sulfate was added to remove the water. After rotary evaporation, the residue was dissolved in 2 mL of acetonitrile and filtered with a 0.22 μm membrane to get the thaxtomin solution. Liquid chromatography with tandem mass spectrometry (4000 QTRAP; AB SCIEX, Framingham, MA, USA) was used to quantify the thaxtomin content in solution with 0.3 mL min^−1^ flow rate of an isocratic mobile phase of 40:60 acetonitrile/water, both of them containing 2 mM ammonium acetate and 0.1% acetic acid. Thaxtomin was detected according to the method described previously [27]. The mycelia of the culture strains were dried and weighed. After dividing the total thaxtomin production by the dry weight of strains, the average thaxtomin production of culture strains was obtained. All experiments were repeated three times with different biological samples of *Streptomyces* strains.

### 2.8. Transcriptomic and RT-qPCR Analyses

*Streptomyces* sp. AMCC400023 was cultured in TDMc medium that contained 7 g L^−1^ of cellobiose. Based on the cell growth and thaxtomin accumulation curves, we collected samples from 36 h, 72 h, and 120 h, with three biological replicates in each group. Total RNA was isolated by using TRIzol RNA extraction kit (Thermo Fisher Scientific, New York, NY, USA) according to the manufacturer’s protocol. Assessment of the RNA integrity was performed on the Agilent 4200 Tape Station (Agilent, Santa Clara, CA, USA); meanwhile, RNA concentration and purity were measured on the Thermo NanoDrop One (Thermo Fisher Scientific). Epicentre Ribo-Zero rRNA Removal Kit was used to remove the ribosomal RNA, and the cDNA libraries were constructed according to the protocol of Illumina’s NEBNextő Ultra II Directional RNA Library Prep Kit and sequenced using Illumina HiSeq 2500 (Illumina, San Diego, CA, USA).

The quality control process used Fastp v0.22.0 software, and clean reads were then mapped to the reference genome using Bowtie2 v2.4.4. FPKM values were calculated for each gene using cufflinks and transformed to TPM values. The DESeq R package was used to identify DEGs with a threshold of *P* < 0.05 and foldChange > 2 or foldChange < 0.5. Heatmap analyses were performed using the complexHeatmap v2.18.0 R package.

The expression levels of *GEO5601* and *GEO7671* were further verified by RT-qPCR, with primers listed in Table S3. For PCR amplification, a total volume of 10 μL containing 5 μL of 2× PerfectStartTM Green qPCR SuperMix, 1 μL cDNA, 0.2 μL gene-specific primer (Table S3), and 3.6 μL ddH_2_O, was used. The program was 30 s at 94 °C, followed by 45 cycles with 5 s at 94 °C, and 30 s at 60 °C. To normalize the gene expression, 16S rDNA was used as an endogenous control. 

## 3. Results

### 3.1. Bioinformatics Analysis of the Cello-Oligosaccharide Transporters

**Figure 1:**
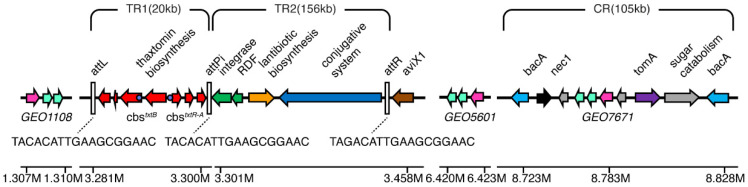
Chromosome position and gene structure maps of three putative CebE sugar transporters and pathogenicity island. The positions and directions of the open reading frames are shown by the arrows. The pink arrows represent the *cebE* genes and the light green arrows represent the *cebF* and *cebG* genes. The red arrows represent the thaxtomin biosynthetic genes, the green arrows represent the integrase and recombination directional factor (RDF), the orange arrow represents the antibiotic synthesis genes, the blue arrow represents the conjugated integration genes, the brown arrow represents the *aviX1* gene, the light blue arrows represent *bacA* genes, the black arrow represents the *nec1* gene, the gray arrows represent the sugar metabolism genes, and the purple arrow represents the *tomA* gene. The att sites used to distinguish toxin region (TR; TR1 and TR2) are also shown. CR stands for colonization region.

The complete genome of *Streptomyces* sp. AMCC400023 was sequenced, annotated, and deposited in the NCBI database under the accession number CP024989. According to the GhostKOALA and eggNOG-mapper annotation results, there are three annotated cello-oligosaccharide binding protein-encoding genes (*cebE*) in the genome, named *GEO1108*, *GEO5601*, and *GEO7671*, respectively. These three *cebE* genes are widely dispersed in the genome (Figure 1); *GEO5601* and *GEO1108* are far away from the TR and CR region, while *GEO7671* is in the CR region of the PAI.

**Figure 2:**
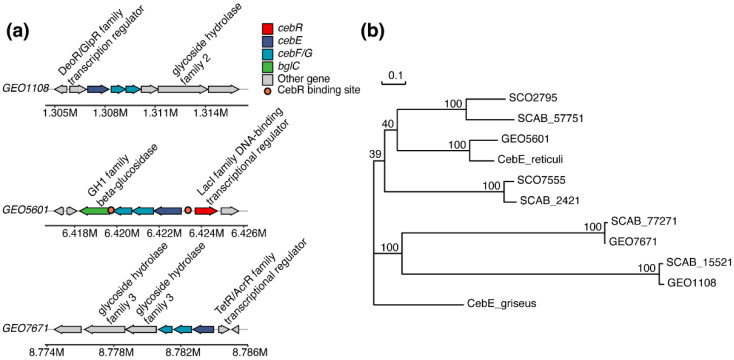
Gene structure of three sugar transporters that anchor the putative cello-oligosaccharide binding gene *cebE* and phylogenetic analysis of CebE orthologues. (**a**) Gene organization of the three cello-oligosaccharide ABC-type transporters. (**b**) NJ tree was constructed with CebE orthologues protein from pathogenic strains: *S. scabies* 87–22 (SCAB_2421: CBG67458.1, SCAB_15521: CBG68694.1, SCAB_57751: CBG72800.1, and SCAB_77271: CBG74696.1), *Streptomyces* sp. AMCC400023 (GEO1108, GEO5601, and GEO7671), nonpathogenic strains: *S. reticuli* (CAB46342), *S. griseus* (WP_012379731.1), and *S. coelicolor* A3 (SCO2795: CAC10104.1, SCO7555: CAC16435.1). Bootstrap values (percentages) are indicated at branching points.

The gene structure of all three annotated transporters follows the typical mode in *Actinobacteria* strains, with CebE, CebF, and CebG, as well as the adjacent glycoside hydrolase (BglC) and a transcription regulator from different families (CebR) included (Figure 2a). All three putative *cebE*s were respectively clustered with one or two glycoside hydrolases, indicating their potential involvement in sugar transport. The glycoside hydrolase gene clustered with *GEO1108* was annotated as beta-galactosidase, while glycoside hydrolases in *GEO5601* and *GEO7671* clusters were both annotated as beta-glucosidases, implying that *GEO1108* may not function as a cello-oligosaccharide binding protein in *Streptomyces* sp. AMCC400023. When searching for the binding sites of CebR regulator, two binding sites were identified in the *GEO5601* gene cluster (TGGGAGCGCTCCCA and TGGAAGCGCTCCCA), while no such *cis*-acting element was found within the *GEO7671* cluster, as observed in its homolog protein SCAB77271 of strain 87–22 [26]. As for the *GEO1108* gene cluster, no cis-acting element was observed. The gene cluster for thaxtomin synthesis contains two CebR binding sites, which is consistent with the presence of *cbs^txtB^* (GGGGAGCGCTCCCA) and *cbs^txtR-A^* (CGGGAGCGCTCCCA) in strain *S. scabiei* 87–22 [24].

Based on the phylogenetic analysis, these three putative CebE sequences are separately distributed into different branches, in which CebE^5601^ is most closely related to CebE*^reti^* (79.05% amino acid sequence identity) that has been previously recognized for its cellobiose-binding ability (Figure 2b). Also, with 46.06% and 46.93% sequence identity, it is closely related to the CebE homologs SCAB2421 and SCAB57751 in strain *S. scabiei* 87–22 (Table S1). The PAI-located CebE^7671^ is clustered with the third CebE sequence (SCAB77271) of *S. scabiei* 87–22, both of which are located in another subtree, with high sequence identity up to 98.8%.

### 3.2. Cellobiose/Cellotriose Affinities of Three CebE Proteins

**Figure 3:**
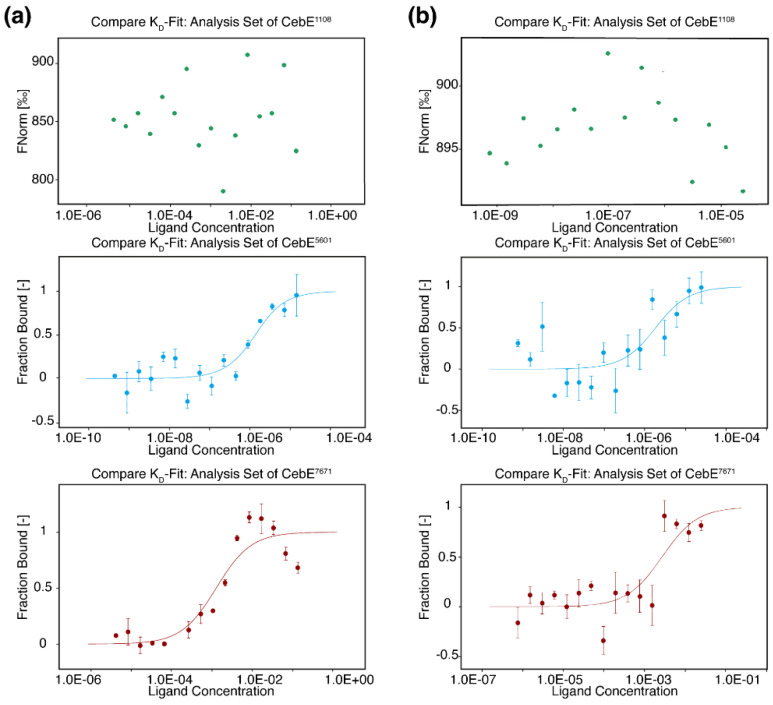
Binding curves of three CebE proteins with cellobiose (**a**) and cellotriose (**b**) by MST assay. The final concentration of CebE was 800 nM. The cellobiose and cellotriose solutions were two-fold diluted 16 times, with an initial concentration of 100 μΜ for CebE^5601^ and 270 mM for GebE^7671^. The *K*_D_ value was determined by MO.Affinity Analysis v2.3 software. Experiments were conducted in triplicate.

To assess the binding affinity to the cello-oligosaccharide substrates, *GEO1108*, *GEO5601*, and *GEO7671* were heterologously expressed and purified (Figure S1) for MST assays. After titration experiments using varying cellobiose concentrations, we obtained *K*_D_ values of 977.19 nM for CebE^5601^ and a value of 1.28 mM for CebE^7671^, which is three orders of magnitude higher (Figure 3a). The micromolar level *K*_D_ value of CebE^5601^ is consistent with its close homolog CebE*^reti^* (1.5 µM) but remarkably higher than that of SCAB57751 (14.2 nM) as measured by the equilibrium dialysis assay or tryptophan intrinsic fluorescence assays [20,25]. However, as different measurement methods were used, the binding differences among these homologs remain to be further evaluated under identical conditions. When switched to cellotriose as the ligand, similar binding affinities were observed as those of cellobiose, with *K*_D_ values of 1.61 μM for CebE^5601^ and 2.72 mM for CebE^7671^ (Figure 3b). In agreement with the clustered beta-galactosidase encoding gene of *GEO1108*, no binding activity was detected between ligands cellobiose/cellotriose and CebE^1108^. Therefore, CebE^1108^ should be assigned as a binding protein for other sugars than cellobiose/cellotriose, and was not considered for further study. Of note, possibly due to strain differences, the indiscriminate binding affinity of cellobiose/cellotriose in the strain we investigated is different from those CebE results of strain 87–22, in which SCAB57751 has a stronger binding affinity with cellotriose than cellobiose [25].

### 3.3. Pathogenicity Assay and Thaxtomin Production of CebE Mutants

**Figure 4:**
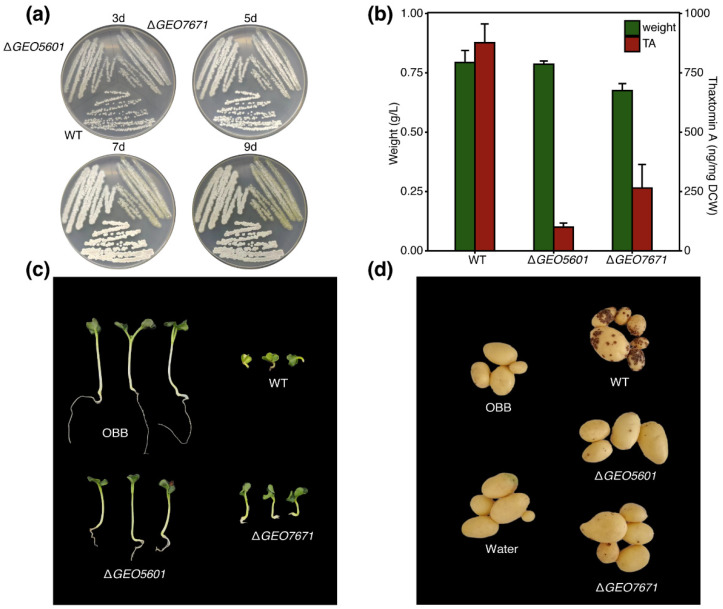
Pathogenicity assays, biomass and thaxtomin production, and morphology of wild-type strain and two CebE mutants. (**a**) The morphology of wild-type strain and mutants on TDMc solid medium. (**b**) Biomass and thaxtomin production of the wild-type strain and mutants. (**c**) Radish seedling bioassay. (**d**) Pot experiment.

To access the contributions of CebE^5601^ and CebE^7671^ to the pathogenic phenotype and thaxtomin production, we successfully constructed the in-frame deletion mutants of Δ*GEO5601* and Δ*GEO7671* for the pathogenicity analysis, with 688 bp and 1063 bp fragments deleted, respectively (Figure S2). To investigate the influence of CebE’s absence on cell growth, the wild-type strain and two mutants were cultured on TDMc medium [16]. After 3-d incubation, the wild-type strain developed intact and regular colonies and quickly produced many spores during 9-d incubations. However, very few spores appeared in Δ*GEO7671* (Figure 4a), even when we prolonged the incubation time to 9 d, implying that CebE^7671^ may assume special functions of mediating spore formation in strain *Streptomyces* sp. AMCC400023. Furthermore, we compared the cell biomass of three strains after culturing in TDMc liquid medium and observed a decreased biomass in strain Δ*GEO7671* (16.4% less than that of the wild-type strain). As for Δ*GEO5601*, growth in solid and liquid medium was relatively stable and only a 5% reduction of the biomass was observed in liquid medium (Figure 4b). Thaxtomin production by two mutants was further compared with the wild-type strain in OBB medium under the same conditions. As a result, abundant thaxtomin was accumulated in strain *Streptomyces* sp. AMCC400023, with the highest detected level of production of 877.09 µg g^−1^. In contrast, the absence of *GEO5601* resulted in a remarkable decrease in thaxtomin synthesis, with only 85.01 µg g^−1^ detected in mutant Δ*GEO5601*. Mutant Δ*GEO7671* showed a modest reduction in thaxtomin synthesis and produced 312.63 µg g^−1^ of it in cultures of 7-d incubation (Figure 4b). We suspect that such reduction may be attributed to the impaired growth of this mutant, which thereby influenced TA synthesis.

The radish seedling bioassay was further performed to compare the pathogenicity of the wild-type *Streptomyces* sp. AMCC400023 and two CebE mutants. As shown in Figure 4c, compared to the strong and well-developed seedlings inoculated with the OBB medium, infection of radish seedlings by wild-type *Streptomyces* sp. AMCC400023 induced typical pathogenic symptoms, including tissue swelling and necrosis, as well as severe stunting of the roots and shoots (Figure 4c). Meanwhile, the infection symptoms of Δ*GEO7671* were also serious, but with somewhat alleviated virulence in the radish seedlings. By contrast, seedlings infected with Δ*GEO5601* exhibited significant differences from the wild-type strain, with obviously reduced tissue necrosis and root and shoot stunting (Figure S3). In agreement with the radish seedling bioassay, the treatment of wild-type *Streptomyces* sp. AMCC400023 also caused severe potato common scab disease, with disease incidence and disease grade up to 97.06% and 63.53%, respectively (Table 2). In contrast to the high pathogenicity of *Streptomyces* sp. AMCC400023, the control treatments of OBB medium and sterile distilled water did not show any scab symptoms (Figure 4d). Compared with the wild-type AMCC400023, the CebE-deficient strains, especially Δ*GEO5601,* obtained remarkably reduced disease indexes. The disease incidence and disease grade caused by Δ*GEO5601* reduced to 37.50% and 11.25%, and a modest disease incidence (70.37%) and disease grade (16.30%) were observed in Δ*GEO7671*. These results matched well with the radish seeding bioassay, suggesting that both CebE^5601^ and CebE^7671^ contribute to the plant-pathogenic phenotype, with CebE^5601^ being the more significant.

### 3.4. Transcriptional Variations of CebE Genes in the Presence of Cellobiose

**Figure 5:**
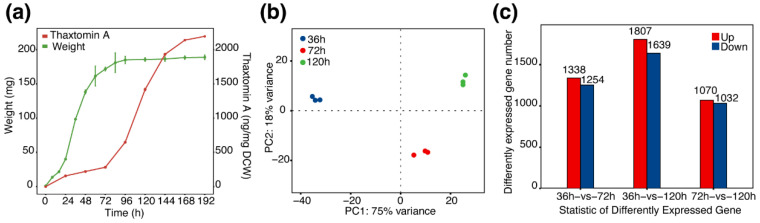
Transcriptomic analysis of *Streptomyces* sp. AMCC400023. (**a**) Growth curve and thaxtomin production of wild-type *Streptomyces* sp. AMCC400023. (**b**) PCA analysis of transcriptome data between treatments. (**c**) A bar plot displaying the numbers of DEGs from the transcriptome data.

To further evaluate the contributions of CebE^5601^ and CebE^7671^ to the nutrient consumption and virulence of *Streptomyces* sp. AMCC400023, the strain was cultured in TDMc and subjected to transcriptomic analysis. As shown in Figure 5a, the growth of strain AMCC400023 exhibited a typical S-curve, with exponential growth after 24 h incubation and entering the stationary phase after 96 h. However, thaxtomin accumulation exhibited a growth-uncoupled mode, with minor thaxtomin detected during the growth phase, while rapid accumulation of thaxtomin occurred after 96h cultivation. Therefore, we sampled the cultures at 36, 72, and 120 h for transcriptomic analysis, with three replicates per treatment. As a result, a total of 56,484,073 clean reads from 9 samples were obtained after quality assessment and data filtering, with an average mapping rate of 96.16% to the reference genome *Streptomyces* sp. AMCC400023 (CP024989) (Table S2). As shown in Figure 5b, the principal component analysis (PCA) shows that samples from three groups were distinguished, with the 36 h group being more dispersed from the 72 h and 120 h groups. Such distribution is reasonable, as the 36 h samples were collected during the early stage of the exponential phase of cell growth, while samples from 72 h were in the latter stage of the exponential phase, and 120 h samples were from the stationary phase. Due to the special phase for thaxtomin synthesis, there were more distinctly expressed genes in the 120 h samples than in the two other groups. Pairwise comparisons of 36 h vs. 72 h, 36 h vs. 120 h, and 72 h vs. 120 h showed their differentially expressed genes (DEGs) were 2592 (1338 up-regulated and 1254 down-regulated), 3446 (1807 up-regulated and 1639 down-regulated), and 2102 (1070 up-regulated and 1032 down-regulated), respectively (Figure 5c).

**Figure 6:**
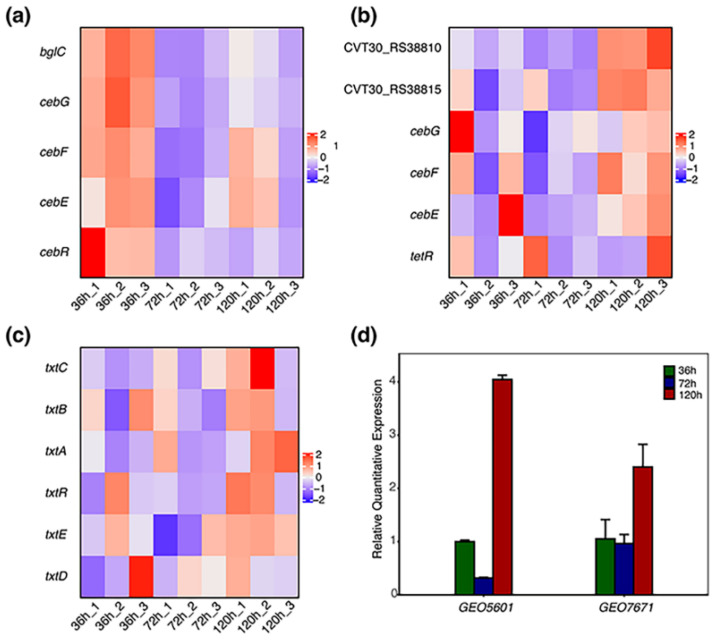
Transcriptional responses of two CebE transporters and thaxtomin biosynthetic clusters in the presence of cellobiose. (**a**) Heatmap of *GEO5601* ABC transporter genes. (**b**) Heatmap of *GEO7671* ABC transporter genes. (**c**) Heatmap of thaxtomin biosynthetic genes. (**d**) Relative expression levels of *GEO5601* and *GEO7671* in RT-qPCR.

The expression heatmap related to *GEO5601* and *GEO7671* gene clusters, as well as the thaxtomin biosynthetic gene cluster, was calculated using the Transcripts Per Million (TPM) values. As the operon, these three clusters are all co-transcribed in all samples and showed regular transcriptional variations during the 120 h cultivation. When comparing the overall expression of two CebE clusters, the *GEO5601* gene cluster displayed relatively high expression in all nine samples. Interestingly, except for the LacI transcriptional regulator that merely upregulated in the 36 h samples, the expression of the other four genes in this cluster all exhibited a trend of ascending after descending (Figure 6a), with the high expressions at 36 h and 120 h and low at 72 h. Compared with the high expressions of *GEO5601* cluster genes, six genes in the *GEO7671* cluster all exhibited relatively low expression during three sampling periods. However, obvious upregulations of all these genes were observed after incubation for 120 h, a period in which thaxtomin accumulated rapidly (Figure 6b). Being resident in the PAI region, the overall expression of the thaxtomin biosynthetic genes was similar to those of *GEO7671* cluster genes, with low expression but upregulated in 120 h samples (Figure 6c). The expression levels of *GEO5601* and *GEO7671* were further verified by RT-qPCR and obtained similar expression profiles (Figure 6d). Well consistent with the transcriptomic data, *GEO5601* also displayed a significantly higher expression level than *GEO7671*. The expression value of 36 h samples was set to 1. The expression level of *GEO5601* showed around 3-fold downregulation in the 72 h samples, then significantly upregulated in 120 h samples, which reached a 4-fold increase. In contrast, the expressions of *GEO7671* remained lower and constant at 72 h, while 2.4-fold upregulation was observed in the 120 h samples.

## 4. Discussion

Pathogenic *Streptomyces* strains generally have two or three sets of *cebR-cebEFG-bglC* gene clusters for cello-oligosaccharides transport. As the signal receptor, the solute-binding protein CebE captures the external signal and functions as the doorway to trigger the regulation network of pathogen virulence [25]. In the well-studied strain *S. scabiei* 87–22, three putative CebEs were identified and their functions for the transporting of cellobiose/cellotriose were verified. Apart from some non-pathogenic *Streptomyces* species, the second CebE (*SCAB2421*) seems only present in the pathogenic *S. scabiei* [26]. In this pathogenic strain, two CebE homologs of SCAB57751 and SCAB2421 both serve as the signal receptors, with SCAB57751 being the most important one. Based on the transcriptional analysis and mutant virulence assay, the PAI-localized *SCAB77271* did not show significant physiological function.

Unlike strain *S. scabiei* 87–22, *Streptomyces* sp. AMCC400023 employs two sets of transporters for uptake of cello-oligosaccharides. Being most closely related to the functional important proteins SCAB57751 and CebE*^reti^*, we suspected that CebE^5601^ is more likely to fulfill the major biological functions of cellobiose/cellotriose recognition and binding in strain *Streptomyces* sp. AMCC400023. Experimentally, several lines of proof confirmed our speculation for the central status of CebE^5601^ in binding cello-oligosaccharides, not only for nutrient consumption but also as a signal in triggering thaxtomin synthesis and pathogenic virulence.

Firstly, CebE^5601^ showed much higher binding affinities toward cellobiose/cellotriose in the MST assay, whereas CebE^7671^ had more than 1000-fold higher *K*_D_ value. Maybe due to the assay bias of different methods or protein difference, when comparing the binding affinity of SCAB57751 that was measured by the tryptophan intrinsic fluorescence, a difference of 2–3 magnitudes existed between SCAB57751 and CebE^5601^ [25]. Also, in *S. scabiei* 87–22, SCAB57751 showed an apparent binding preference towards cellotriose but not cellobiose (2.1 nM and 14.2 nM, respectively). It was supposed that the higher affinity to cellotriose could be a key adaptation for this bacterium to perceive cellotriose as the major signal for triggering thaxtomin synthesis instead of a nutrient uptake response [25]. By contrast, and worth noticing, no apparent difference in binding values between cellobiose and cellotriose was observed for the CebE^5601^ protein of *Streptomyces* sp. AMCC400023. Species differences would be an applicable explanation for such affinity and need to be further addressed.

Secondly, as proven by transcriptomic and RT-qPCR analyses, the *cebE* clusters of *GEO5601* are significantly more highly expressed throughout the cultivation process in TDMc liquid medium. Also, compared to the later exponential period (72 h), *GEO5601* was highly upregulated during the midst of the exponential phase when the strain grew vigorously (36 h). In combination with the constant and low expressions of the *GEO7671* gene cluster, we propose that the *GEO5601* transport system is functional in cellobiose uptake and substantially contributes to the nutrient consumption of the cells. After the later exponential period, the restored expression of *GEO5601* in the 120 h samples further confirmed its functions in thaxtomin synthesis. This result is partly consistent with the findings in strains *S. scabiei* 87–22 and *S. scabiei* EF-35. When grown in the minimal starch medium supplement with 5 g L^−1^ of cellobiose, the expressions of *SCAB2421,* especially *SCAB57751,* were both upregulated [31,32].

Thirdly, in-frame deletions of two *cebE* genes resulted in different responses in plant infection. Through radish seedling assay, we found that the growth of the seedlings infected by two CebE mutants was better than that of the wild-type strain, and both showed alleviated pathogenic symptoms, which preliminarily indicated that the knocking-out of the *cebE* did have an impact on pathogenicity. Remarkably, the seedling growth of ∆*GEO5601* was better than that of ∆*GEO7671*, implying that CebE^5601^ might be more important for its pathogenicity. As radish seedling assay can only partly reflect the pathogenicity of *Streptomyces* and the whole infection process is more complicated, we further performed the potato pot assay. Consistent with these results, the thaxtomin production of ∆*GEO7671* did not change significantly compared with that of the wild-type strain, but the thaxtomin production of ∆*GEO5601* showed a significant decrease, with only 11% of the wild-type yield retained.

**Figure 7:**
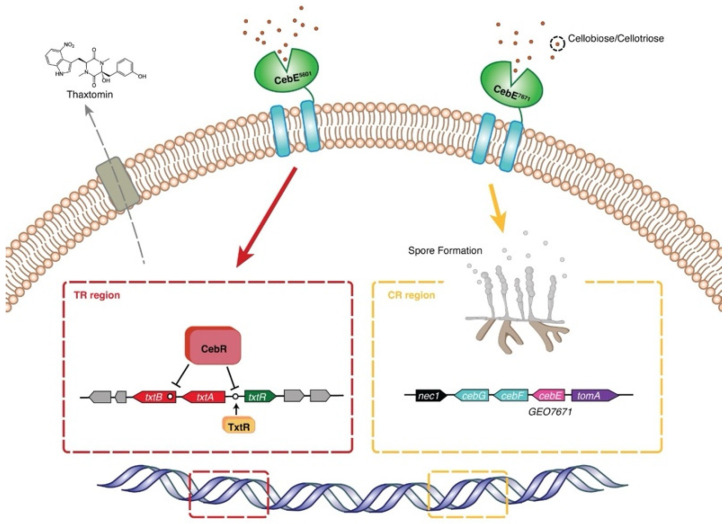
Schematic of the collaborative mode of two CebEs in *Streptomyces* sp. AMCC400023. CebE^5601^ and CebE^7671^ are anchored and separately distributed on the lipid membrane. CebE^5601^ serves as the protagonist and invokes the synthesis of arsenal thaxtomin by repressively binding with CebR to activate the transcriptional activator TxtR. CebE^7671^ not only participates in the signal transfer for thaxtomin synthesis, but also assumes a novel and regulative function of sporulation that may be significant to the host colonization of the pathogen.

In addition to the central status of CebE^5601^ in cello-oligosaccharides transport and strain virulence, the specific location (PAI) of *GEO7671* makes it an attractive target to explore its physiological functions in pathogenic *Streptomyces* strains. With weak binding affinities to cellobiose/cellotriose, the seriously impaired spore formation of Δ*GEO7671* implies that it might be involved in the spore development process. Meanwhile, the absence of CebE^7671^ modestly impaired cell growth and consequently alleviated the pathogenicity of *Streptomyces* sp. AMCC400023. In combination with the 4.1-fold upregulation of *GEO7671* in the late growth stage (120 h), we propose that this PAI-located CebE transporter may also be involved in strain virulence, maybe not for signal reception, but is functional in the sporulation and thus makes it conducive to occupying a specific ecological niche. In the identified *GEO7671* gene cluster (Figure 2a), a regulator of the TexR/AcrR family is adjacent to the *GEO7671* gene. Referring to the finding that a TexR/AcrR regulator in *S. coelicolor* mediates the development of aerial hyphae and spores, we suspect that this gene regulation would be an explanation for the impaired sporulation of Δ*GEO7671* and needs to be further elucidated [33,34]. Integrating the physiological roles of CebE^5601^ and CebE^7671^, we propose that the pathogenic *Streptomyces* sp. AMCC400023 relies on two sets of CebEFG-BglC transporters for growth and pathogenicity, with distinct contributions to strain colonization, growth dynamics, and virulence (Figure 7). Specifically, under cellobiose/cellotriose signals, CebE^5601^ plays a pivotal role in sugar uptake, nutrient consumption, thaxtomin synthesis, and infection symptom manifestation. Meanwhile, CebE^7671^ assumes a novel biophysical function of spore formation and potentially contributes to the colonization of expanding plant tissue.

## 5. Conclusions

Investigating the physiological function of CebE is of utmost significance due to its pivotal role as the recipient of cello-oligosaccharides, not only in sugar transport but also as a signal for strain virulence. In this study, we have discovered a novel mechanism of two CebE systems in the pathogenic *Streptomyces* sp. AMCC400023. The unique presence of PAI-localized CebE^7671^ for sporulation suggests that diverse mechanisms may exist in various pathogenic *Streptomyces* strains, and thereby provide additional targets for drug development against potato common scab.

## Figures and Tables

**Table 1:** Bacterial strains and plasmids used in this study.

	Descriptions
**Plasmids**	
pJTU1278	Shuttle plasmid for DNA transfer (Km, Amp, Cml)
pEASY-Blunt	Cloning plasmid (Amp)
pET28a	Expression plasmid used to produce N-terminal His-tagged protein (Km)
**Strains**	
*E. coli* DH5α	General cloning host
*E. coli* BL21(C43)	Host for protein expression
*E. coli* ET12567	Host for transfer of DNA into *Streptomyces* spp.
*Streptomyces* sp. AMCC400023	Wild type strain
Δ*GEO5601*	AMCC400023 derivative with *GEO5601* deficient
Δ*GEO7671*	AMCC400023 derivative with *GEO7671* deficient

**Table 2:** Morbidity of pot assay.

Treatment	Incidence Rate (%)	Disease Index (%)
CK1-Water	-	-
CK2-OBB	-	-
T1-WT	97.06	63.53
T2-Δ*GEO5601*	37.50	11.25
T2-Δ*GEO7671*	70.37	16.30

## Data Availability

The genomic sequencing data of *Streptomyces* sp. AMCC400023 was deposited in the NCBI database with accession number CP024989. Transcriptomic sequencing data of samples were deposited in the NCBI Short Read Archive (SRA) database under Bioproject accession number PRJNA419158, with accession numbers SRR26158258-26158294.

## Supplementary Materials

The following supporting information can be downloaded at: https://www.mdpi.com/article/10.3390/microorganisms12030499/s1, Figure S1: SDS-PAGE spectrums of the purification process of three putative CebE protein; Figure S2: PCR verification of the in-frame deletion *Streptomyces* mutants; Figure S3: The significance analysis of radish seedling bioassay; Table S1: The amino acid sequence identity of protein used in the phylogenetic tree; Table S2: Summary of sequencing and assembly results for the RNA samples; Table S3: Primers used in this study.
