# Supplementary material for: Physiological Functions of the Cello-Oligosaccharides Binding CebE in the Pathogenic Streptomyces sp. AMCC400023
Source: Microorganisms. 2024 Feb 29;12(3):499. doi: 10.3390/microorganisms12030499 (PMC10974276; doi:10.3390/microorganisms12030499)
Supplement: Supplementary file 1 [file microorganisms-12-00499-s001.zip › microorganisms-2876554-supplementary.pdf]

SUPPLEMENTARY INFORMATION

SUPPLEMENTARY TABLES AND FIGURES

TABLE S1. The amino acid sequence identity of protein used in the phylogenetic tree.

	**CebE_griseus**	**CebE_reticuli**	**SCO2795**	**SCO7555**	**SCAB_2421**	**SCAB_15521**	**SCAB_57751**	**SCAB_77271**	**GEO1108**	**GEO5601**	**GEO7671**
**CebE_griseus**	100	47.99	46.28	49.4	47.79	34.55	47.09	39.76	34.47	48,05	39.76
**CebE_reticuli**		1000	51.38	45.61	45.75	32.51	46.83	36.02	32.05	79.05	36.56
**SCO2795**			100	47.95	47.21	29.41	71.39	33.48	29.19	51.58	34.8
**SCO7555**				100	90.59	33.1	47.46	36.22	33.1	45.25	35.95
**SCAB_2421**					100	32.33	46.19	35.14	32.08	46.06	35.41
**SCAB_15521**						100	30.47	29.92	96.48	34.1	29.66
**SCAB_57751**							100	35.14	29.17	46.93	34.64
**SCAB_77271**								100	30.31	36.05	98.8
**GEO1108**									100	33.9	30.18
**GEO5601**										100	36.79
**GEO7671**											100

TABLE S2. Summary of sequencing and assembly results for the RNA samples.

Sample	Clean Reads	Q30 (%)	Mapping Ratio
36h-1	6313294	96.17%	95.46%
36h-2	6842781	96.13%	97.94%
36h-3	5436705	96.26%	97.72%
72h-1	4276641	96.21%	96.72%
72h-2	6796329	96.38%	94.90%
72h-3	4974938	96.19%	96.43%
120h-1	6549152	96.05%	93.73%
120h-2	6307468	96.10%	94.60%
120h-3	8986765	96.39%	98.00%

TABLE S3. Primers used in this study. The restriction sites are underlined.

Primers	Sequence (5′→3′)	Description
1108F (*Nde*I)	CCCATATGGTGGGCTCGGATTCC	Gene Cloning
1108R (*Xho*I)	CCCTCGAGTCAGTTCACCCCCT	
5601F (*Nde*I)	CCCATATGATGGACGGCGGCTC	
5601R (*Bam*HI)	CGGGATCCTCACTGGCCGAGCACGT	
7671F (*Nde*I)	CCCATATGATGGAGGGCGATGC	
7671R (*Xho*I)	CCCTCGAGTCACTTCAGCGAGTCCA	
5601①F	CGGGATCCTCGGCTCCGGCGCCCGCTGC	Gene Knockout
5601①R	GGAGGACGCACCAGCTCCAAAGAGGCACTC	
5601②F	TTGGAGCTGGTGCGTCCTCCTGATGCCC	
5601②R	CCAAGCTTCAGATCCGGGAGCGGGTCGT	
7671①F	CGGGATCCCTGCGCGACCTCTGCCGACGT	
7671①R	ACTGGGACCTCATCGGTGGACAGGGCGAATT	
7671②F	TCCACCGATGAGGTCCCAGTCGTGCAGC	
7671②R	CCAAGCTTATGGCCTCGACGACATC	
5601①VF	ACAGCAGCACGAACACCAGC	Knockout Verification
5601①VR	CAGCCCGATAACCGAAGACA	
7671①VF	CGAGGATGAAGGTGAAGTTGTC	
7671①VR	TCTGGGCTGCCTGGTAGTGC	
5601F	CCTTCGGAGTCTTCGGCTA	qRT-PCR
5601R	AGTAGACCGAGGTCTTGG	
7671F	GACCAGTACGAGGTGCTCTG	
7671R	TCGTAGATGTTCCGGAGGTC	

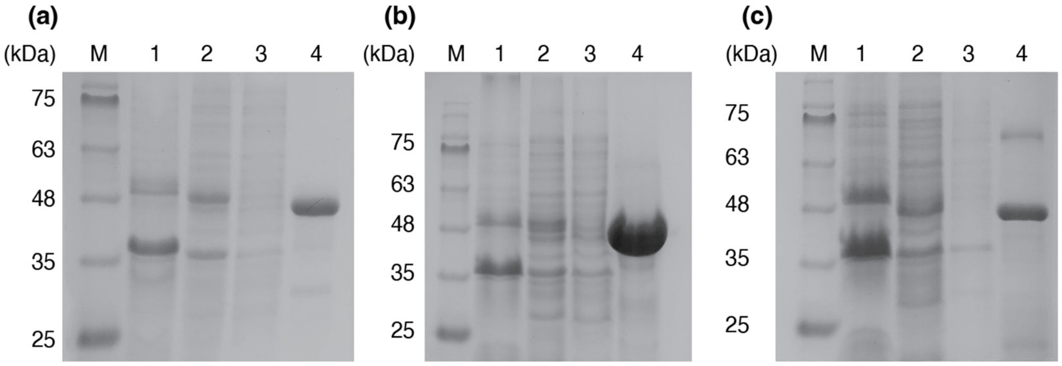

FIGURE S1 SDS-PAGE spectrums of the purification process of three putative CebE protein of $CebE^{1108}$ (**a**), $CebE^{5601}$ (**b**), and $CebE^{7671}$ (**c**). Lane M, marker; Lane 1, crude enzyme extract; Lane 2, inclusion body; Lane 3, effluent from the washing process; Lane 4, elution by 500 mM imidazole.

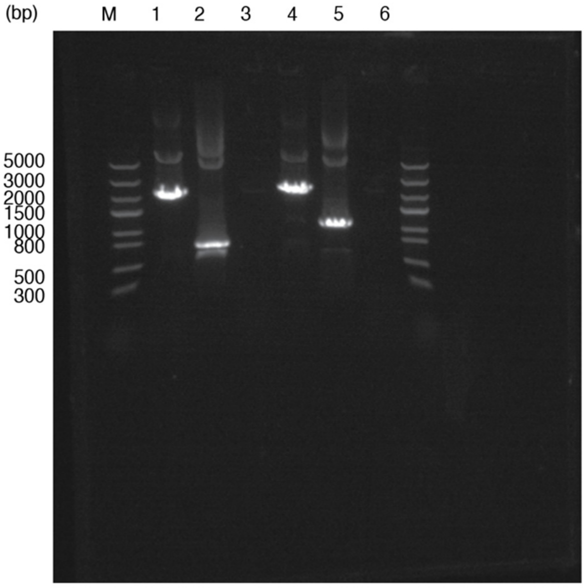

FIGURE S2 PCR verification of the in-frame deletion *Streptomyces* mutants Δ*GEO5601* and Δ*GEO7671*. Lane M, marker; Lane 1, positive control of *GEO5601* (2046 bp); Lane 2, mutant that only carrying the deleted *GEO5601* fragment (688 bp); Lane 3, negative control with no template supplementation; Lane 4, positive control of *GEO7671* (2272 bp); Lane 5, mutant that only carrying the deleted *GEO7671* fragment (1063 bp); Lne 6, negative control with no template supplementation.

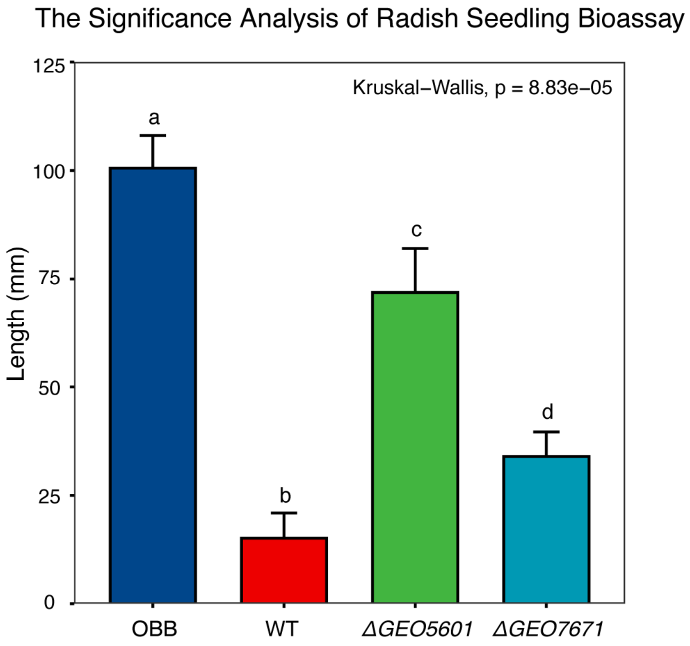

FIGURE S3 The significance analysis of radish seedling bioassay.
